# No Apparent Reduction in Schistosome Burden or Genetic Diversity Following Four Years of School-Based Mass Drug Administration in Mwea, Central Kenya, a Heavy Transmission Area

**DOI:** 10.1371/journal.pntd.0003221

**Published:** 2014-10-09

**Authors:** Agola E. Lelo, David N. Mburu, Gabriel N. Magoma, Ben N. Mungai, Jimmy H. Kihara, Ibrahim N. Mwangi, Geoffrey M. Maina, Joseph M. Kinuthia, Martin W. Mutuku, Eric S. Loker, Gerald M. Mkoji, Michelle L. Steinauer

**Affiliations:** 1 Centre for Biotechnology Research and Development, Kenya Medical Research Institute (KEMRI), Nairobi, Kenya; 2 Kenyatta University, Department of Biochemistry, Nairobi, Kenya; 3 Department of Biochemistry, Jomo Kenyatta University of Agriculture and Technology (JKUAT), Nairobi, Kenya; 4 Department of Biology, University of New Mexico, Albuquerque, New Mexico, United States of America; 5 Department of Basic Medical Sciences, College of Osteopathic Medicine of the Pacific Northwest, Western University of Health Sciences, Lebanon, Oregon, United States of America; Swiss Tropical and Public Health Institute, Switzerland

## Abstract

**Background:**

Schistosomiasis is a debilitating neglected tropical disease that infects over 200 million people worldwide. To combat this disease, in 2012, the World Health Organization announced a goal of reducing and eliminating transmission of schistosomes. Current control focuses primarily on mass drug administration (MDA). Therefore, we monitored transmission of *Schistosoma mansoni* via fecal egg counts and genetic markers in a typical school based MDA setting to ascertain the actual impacts of MDA on the targeted schistosome population.

**Methods:**

For 4 years, we followed 67 children enrolled in a MDA program in Kenya. Infection status and egg counts were measured each year prior to treatment. For 15 of these children, for which there was no evidence of acquired resistance, meaning they became re-infected following each treatment, we collected microsatellite genotype data from schistosomes passed in fecal samples as a representation of the force of transmission between drug treatments. We genotyped a total of 4938 parasites from these children, with an average of 329.2 parasites per child for the entire study, and an average of 82.3 parasites per child per annual examination. We compared prevalence, egg counts, and genetic measures including allelic richness, gene diversity (expected heterozygosity), adult worm burdens and effective number of breeders among time points to search for evidence for a change in transmission or schistosome populations during the MDA program.

**Findings:**

We found no evidence of reduced transmission or schistosome population decline over the course of the program. Although prevalence declined in the 67 children as it did in the overall program, reinfection rates were high, and for the 15 children studied in detail, schistosome egg counts and estimated adult worm burdens did not decline between years 1 and 4, and genetic diversity increased over the course of drug treatment.

**Interpretation:**

School based control programs undoubtedly improve the health of individuals; however, our data show that in an endemic area, such a program has had no obvious effect on reducing transmission or of significantly impacting the schistosome population as sampled by the children we studied in depth. Results like these, in combination with other sources of information, suggest more integrated approaches for interrupting transmission and significantly diminishing schistosome populations will be required to achieve sustainable control.

## Author Summary

Schistosomiasis is a chronic and debilitating disease. Current control focuses primarily on mass drug administration (MDA). We monitored schistosome transmission via fecal egg counts and genetic markers in a school based MDA setting to learn how the intervention was influencing transmission or the targeted schistosome population. For 4 years, we followed 67 children enrolled in a MDA program in Kenya, and for 15 of these, we repeatedly acquired in depth genetic data regarding the schistosome populations they harbored. Although prevalence declined in the 67 children, we found no evidence of reduced egg counts/worm burdens in those that reacquired infections and genetic diversity of schistosomes increased over the course of treatment. Our data indicate that this school based MDA program had a strong benefit to individual health as fewer children were infected over time. However, this decline does not appear to be due to schistosome population reduction, and may be caused by either acquired resistance or behavioral changes of the children. In conclusion, control programs based on chemotherapy alone or based on only a subset of the population will need to be supplemented with additional approaches if we are to achieve the WHO goal of eliminating human schistosomiasis by 2025.

## Introduction

Recently, there has been increased awareness of the massive global health burden of schistosomiasis and other neglected tropical diseases (NTDs). Notably, in 2001, Resolution 19 of the World Health Assembly called for increased drug treatment of NTDs to reach a minimum coverage of 75% of school aged children in endemic areas (WHA 54.19). Thus, the initiation of multiple mass drug administration (MDA) programs was prompted [1]. School aged children have been specifically targeted because they are suspected to harbor the heaviest worm burdens and thus experience a high degree of morbidity from infection. Additionally, school systems provide infrastructure within which such programs can successfully operate, a key consideration for lowering the cost of drug administration.

Traditionally, the primary goal of MDA programs has been to reduce worm burdens in individuals and thus reduce the morbidity caused by NTDs. However, a revised roadmap published by the World Health Organization in 2012 reaches further: toward the elimination of NTD transmission in some regions [2]. To a large part, this goal is being facilitated by a major increase of pharmaceutical donations to expand coverage of MDA. With this goal in mind, it is important to determine if current deworming programs are moving toward elimination by reducing schistosome transmission in endemic regions where MDA programs are ongoing. This knowledge is critical to inform control protocols to achieve the WHO goals.

It is challenging to determine if drug treatment of a focal group of patients is reducing the entire schistosome population in a region (especially so for worms in untreated people) and thus the force of infection. For schistosomes, the force of infection has been defined as the rate of establishment of patent infections. Ideally, one would treat all infected people; however, this is often not logistically possible. Population genetic estimates indicate that schistosome populations are large and widely distributed, not only geographically but also among snails, human hosts of a wide range of ages and reservoir hosts [3]–[6]. Thus, most MDA programs treat a portion of people infected with schistosomiasis, exposing only a portion of the schistosome population to drug treatment while the rest of the population remains in “refugia”, isolated from drug exposure. Because the goal of most deworming programs is to reduce worm burdens in individuals and thus morbidity (rather than elimination or reducing the force of infection), success is typically assessed by comparing infection intensity (worm burden estimated by fecal egg counts) and prevalence of infection in the treated portion of the population from before treatment to some time point afterwards. It is difficult to use these data to assess the reduction of the larger schistosome population for two reasons. First, infection starts in infancy and schistosomes are long lived (5–10 years) [7]–[9], thus pre-mass drug treatment levels of infection reflect long term accumulation of worms and should be higher than recolonization levels after treatment clearance [10]. Second, it has been shown that a large portion of individuals treated for schistosomiasis acquires partial resistance to reinfection that is measurable within 2–3 drug treatments [11]–[15]. Thus, a decrease in schistosome burdens in the treated portion of the population is expected, and this decrease may not reflect an actual decrease in the entire schistosome population or in the force of infection. In fact, it is possible that the force of infection could actually be increasing even when worm burdens of the treated individuals are declining due to acquired resistance in the treated population.

One way to monitor the change in schistosome populations is to monitor those treated individuals who do not acquire resistance to reinfection after repeated treatments (i.e. those that remain susceptible). Such individuals were termed “phenotypically susceptible” by Black et al. 2010 [11] because they failed to produce protective immunity after repeated drug treatment. Assuming comparisons are not made with pre-control baselines, treatment is successful, and prior worm burdens are cleared, the worm populations acquired by patients after treatment can be measured by the number of schistosome eggs released in a fecal sample via the Kato-Katz methodology [16], [17], especially when coupled with genetic data on miracidia from these eggs. Genetic data can provide an additional and powerful perspective because they can be used to estimate worm burdens within an individual ([i.e. number of breeding pairs represented by sibling groups 18]), to detect overall population declines (i.e. population bottlenecks [19], [20]), and measure genetic diversity which is a measure of the parasite's ability to adapt to environmental pressures [21].

Some attempts have been made to assess changes in schistosome populations following drug treatment. Norton et al. [21]. monitored changes in microsatellite populations of *Schistosoma mansoni* miracidia (n = 20 per child) derived from Tanzanian school children following a single round of treatment and found a significant decline in genetic diversity [21]. They noted this was true even for young children entering the school who had not been treated previously. Following the fate of infections in individual hosts as an important indicator of efficacy of control was also suggested. Using cytochrome c oxidase I as a marker, Betson et al. [22] found substantial genetic diversity of *S. mansoni* within both children less than six years old and their mothers (they examined 1347 parasites from 35 mothers and 45 children) [22]. They did not observe any change in schistosome genetic diversity before or six months after treatment in samples collected from the same individual or from the same family. The authors noted that even exposure times of 1.5 years were sufficient to result in genetically diverse infections in young children.

Within the context of a typical school-based MDA program in central Kenya targeting *S. mansoni* for control on an annual basis, we took an approach that emphasized a deeper sampling of miracidia within individual children, and that began with examination of children for four years following treatment. Deeper sampling enables the calculation of novel measurements of worm burdens within a patient and also enables genetic diversity to be more accurately measured because the bias that is introduced by including related parasites in the sample can be reduced (see Steinauer et al. [23]). We used both fecal egg counts and genetic measures to assess changes in phenotypically susceptible children over a 4 year period (2008–2011).

## Materials and Methods

### Ethical Statement

This protocol was approved by both the Kenya Medical Research Institute (KEMRI) Scientific committee and the KEMRI Ethical committee. Parents/guardians of all children involved provided written informed consent on behalf of all child participants. Informed consent was obtained by first holding a meeting with the parents/guardian to explain the purpose of study followed by a question and answer session. Thereafter, they were requested to sign the written consent forms on behalf of the children.

### Sample collection

A school-based schistosomiasis and soil-transmitted helminth (STH) control project in Mwea, Kenya was established in 2004 through a collaboration between the Japan International Cooperation Agency (JICA) and the Kenya Medical Research Institute (KEMRI). Mwea is a large rice growing region in the Kirinyaga County, central Kenya. The Thiba and Nyamindi Rivers that pass through this area serve as source of water for the irrigation schemes, and schistosomiasis transmission is believed to take place primarily from the irrigation canals that supply water to the rice schemes. A pilot study in 2004 measured prevalence of helminths in school aged children followed by MDA (praziquantel and albendazole) [24]. At this time, the human population of the area was estimated to be 125,000 and the prevalence of *Schistosoma mansoni* in school aged children was 47.4%.

Starting in 2004, the KEMRI-JICA Project administered annual doses of anthelmintics to all school aged children (>40,000) in the region regardless of their infection status. This treatment included a single dose of 40 mg/kg of praziquantel using the tablet dose pole to determine the number of tablets [25] and albendazole in a 400 mg single dose. Prior to the beginning of the program a baseline determination of prevalence and intensity of parasitic infections through examination of stool samples of class three children (age range 5–14 years) was undertaken [26]. Our study recruited the subjects by randomly sampling among the children who had previously tested positive within this cohort. We focused on infected individuals because we were seeking those that were not acquiring resistance to reinfection with repeated drug treatment. Starting in 2008, we followed 67 students previously enrolled in the KEMRI-JICA Project. These students were between 5 and 14 years of age and were from four primary schools: Kirogo (00°39S/37°23E), Nyamindi (00°40 S/37°24E), Mukou (00°40S/37°20E) and Ngurubani (00°41S/37°21E). The schools are less than 8 km apart ([figure in 3]). Prior to each treatment, fecal samples were obtained from three consecutive days and infection was assessed using the Kato Katz methodology [16], [17]. Following each treatment, there was a three month follow-up stool exam for each child, and each child was found to be negative (treatment was successful in every case). For the Kato-Katz procedure, two slides per patient were examined for schistosome eggs.

To assess population level reduction in schistosome transmission, we collected genetic data from the schistosome infrapopulations of 15 of the 67 children. These 15 children were deemed phenotypically susceptible throughout the four year time period because they became reinfected at every time point with large worm burdens (as determined by egg counts) indicating high susceptibility of these children. More of the 67 children also fell into the phenotypically susceptible category, but we only compiled genetic data from the 15 children from whom we were able to collect samples at each sampling period. Miracidia were collected from their fecal samples and used for microsatellite genotyping. Schistosome miracidia were hatched from fecal samples [27] and individuals were genotyped at 12 microsatellite loci [28] and included GenBank accession numbers: *AF325695*, *AF325698*, *AF202965*, *AF202966*, *AF202968*, *L46951*, *M85305*, *R95529*, *AF202968*, *AI395184*, *AI067617* and *BF936409*.

### Data Analysis

We compared reductions in fecal egg counts in the 15 phenotypically susceptible children and in the other 52 children over the 4 time points (annual samples prior to MDA 2008–2011) using a repeated measures ANOVA with Systat 11 (Systat Software, Inc.). Data were natural log transformed to meet the assumptions of parametric statistical tests. Single degree of freedom polynomial contrasts were used to determine the significance of trends in the data over time. The hypothesis was that a reduction in the force of infection of schistosomiasis would be matched with a reduction in fecal egg counts in all children and in the 15 phenotypically susceptible children.

We also compared population genetic parameters across the four time points at two levels: populations within each patient (infrapopulations) ([e.g. 21]) and all patients combined (component population) ([e.g. 10]). We hypothesized that significant reductions in the schistosome population should be accompanied by reductions in all of these genetic parameters over the four year time period.

For the infrapopulation level analysis, we first compared two estimators of the worm burdens within a patient: the number of full sibling families, and the effective number of breeders (N_b_). These parameters were compared across all time points using repeated measures ANOVA for each dependent variable. The number of full sibling families is a measure of the number of breeding worms within a patient (worm burden) because the miracidia collected in a fecal sample are offspring of the adult worms inside the patient. The offspring were partitioned into their families based on shared alleles using COLONY v.2.0 [29], [30]. COLONY has been shown to accurately reproduce schistosome families using genotype data at the same microsatellite loci [18]. N_b_ was estimated using the sibling assignment method implemented in COLONY [31]. N_b_ is the effective population size N_e_ measured from a single breeding cohort (miracidia derived from a single patient).

We also compared two measures of population genetic diversity: allelic richness and gene diversity (expected heterozygosity). We corrected our datasets for the bias induced by related miracidia in a fecal sample by inferring the family structure present in a sample, and then resampling the dataset to include only one member of each family [18], [32]. Ten resampled datasets were generated and both parameters were calculated using FSTAT 2.9.3 [33]. Parameters were natural log transformed and compared across patients between the first and last sample using repeated measures ANOVA (Systat 11, Systat Software, Inc.). One patient was removed from the analysis due to high family structure and resulting low sample size in the resampled datasets (n = 6 unrelated individuals remaining after correction, thus all individuals sampled fell into one of 6 families).

At the component population level (all patients combined within a year), we used permutation tests on the 10 corrected datasets to determine significant differences in gene diversity and allelic richness between year 1 and year 4 of the MDA program. These tests were performed with FSTAT and 10,000 permutations were used to determine significance.

## Results

Table 1 provides a summary of the 67 children investigated, and includes their gender, age at baseline, and pre-treatment egg counts (average eggs per gram of feces determined by Kato-Katz) at each of the four annual treatment periods. Table 2 indicates for the 15 phenotypically susceptible children investigated in depth, for each annual observation, the number of miracidia genotyped (N), the effective number of breeders (N_b_), and the schistosome census number (N_c_). We genotyped a total of 4938 parasites from these children, with an average of 329.2 parasites per child for the entire study, and an average of 82.3 parasites per child per annual examination.

**Table 1:** A summary of the demographic characteristics of 67 children investigated including their gender, age at baseline, and pre-treatment egg counts (average eggs per gram of feces determined by Kato-Katz) at each of the four annual treatment periods.

Sample ID	School	Gender	Age at baseline	Year 1	Year 2	Year 3	Year 4
				*(EPG)*	*(EPG)*	*(EPG)*	*(EPG)*
TB2*	Nyamindi	M	6	4320	6240	624	6144
MK10*	Nyamindi	M	6	1008	864	1656	2976
TB9*	Nyamindi	M	6	480	2784	1656	1296
TB4*	Nyamindi	F	6	9600	1512	2304	8448
NG46	Nyamindi	F	7	48	96	288	0
KB35	Nyamindi	F	7	72	48	192	96
KB81	Nyamindi	F	8	72	0	48	0
NG84	Nyamindi	F	8	69	26	48	3
KR1	Nyamindi	M	8	72	2256	96	24
NG126	Nyamindi	M	8	192	648	0	0
NG11	Nyamindi	F	9	312	288	0	336
NY67	Nyamindi	F	9	912	120	0	0
NY77	Nyamindi	M	11	24	0	0	24
TB15*	Kirogo	M	6	2880	2304	168	1560
TB120*	Kirogo	F	6	9600	1512	96	600
TB6*	Kirogo	M	6	2400	432	216	2304
TB132*	Kirogo	F	6	960	1008	336	792
KB18	Kirogo	M	7	432	0	168	96
NY06	Kirogo	F	7	72	2256	768	96
NY33	Kirogo	F	7	24	240	2256	0
MU1	Kirogo	M	7	2664	336	336	0
KB13	Kirogo	F	7	2784	0	336	0
NY27	Kirogo	F	7	48	0	240	24
NG24	Kirogo	F	7	1032	336	408	120
NY68	Kirogo	F	8	864	120	144	120
NY60	Kirogo	F	8	240	42	120	216
KB19	Kirogo	F	9	0	120	0	0
NG118	Kirogo	F	9	4992	168	0	24
NY14	Kirogo	F	10	288	48	0	264
KB18	Kirogo	M	11	24	0	0	48
TB10*	Ngurubani	F	6	480	192	336	288
TB19*	Ngurubani	M	6	8160	7776	432	768
TB3*	Ngurubani	F	6	1440	1344	4320	6096
NG77	Ngurubani	F	7	72	240	432	0
NG116	Ngurubani	F	8	144	432	0	432
NY76	Ngurubani	M	8	120	768	0	648
NG39	Ngurubani	F	8	72	24	48	0
NG74	Ngurubani	F	8	336	216	26	0
NY49	Ngurubani	F	9	264	288	0	72
KB21	Ngurubani	M	9	0	360	0	0
NY66	Ngurubani	M	10	312	0	0	0
NG71	Ngurubani	F	10	1872	0	0	48
NG110	Ngurubani	M	10	192	48	0	408
NY44	Ngurubani	M	11	96	0	0	0
NG109	Mukou	F	6	72	240	168	1128
TB130*	Mukou	M	6	2400	7680	192	2328
TB14*	Mukou	M	6	2880	432	264	2040
MK4*	Mukou	F	6	1128	2352	2304	3024
MK12*	Mukou	F	6	4320	6360	2304	1320
NG41	Mukou	M	7	264	0	168	0
NY112	Mukou	F	7	369	0	2784	48
M51	Mukou	F	7	24	432	1008	168
NG11	Mukou	M	7	312	192	384	24
NY39	Mukou	M	7	1128	0	432	384
NG33	Mukou	F	7	144	360	264	216
NG45	Mukou	F	7	384	48	192	240
KB67	Mukou	M	8	24	48	24	0
KR56	Mukou	F	8	264	0	48	0
NY15	Mukou	M	8	72	24	72	144
NY78	Mukou	F	8	528	144	72	0
NY34	Mukou	F	8	336	72	96	120
NY35	Mukou	F	8	864	384	120	672
NG128	Mukou	F	9	48	96	0	24
NY39	Mukou	M	9	48	288	0	0
NY45	Mukou	F	9	48	408	0	0
KR38	Mukou	M	10	168	0	0	0
NY17	Mukou	F	10	384	0	0	1872

**Table 2:** Temporal estimates of schistosome burdens of fifteen school aged children enrolled in a mass drug administration program in which they are treated annually.

	Year 1				Year 2				Year 3				Year 4			
Patient	n	N_b_	CI	N_c_	n	N_b_	CI	N_c_	n	N_b_	CI	N_c_	n	N_b_	CI	N_c_
1	96	172	130–233	344–1720	81	20	12–38	40–200	81	158	117–220	316–1580	103	210	160–285	420–2100
2	89	151	114–206	302–1510	68	127	92–182	254–1270	81	120	89–168	240–1200	76	97	72–137	194–970
3	73	99	72–142	198–990	86	149	112–203	298–1490	86	52	36–77	104–520	81	209	149–301	418–2090
4	68	142	103–205	284–1420	73	195	142–289	390–1950	76	154	108–220	308–1540	107	103	75–141	206–1030
5	144	160	125–206	320–1600	81	24	14–43	48–240	103	118	88–160	236–1180	63	122	87–175	244–1220
6	128	153	120–199	306–1530	150	246	195–316	492–2460	139	256	201–334	512–2560	78	98	71–140	196–980
7	58	81	56–118	162–810	57	67	46–100	134–670	83	28	17–49	56–280	60	107	76–160	214–1070
8	68	130	93–186	260–1300	60	136	95–200	272–1360	53	172	113–298	344–1720	85	117	88–161	234–1170
9	58	75	51–112	150–750	68	114	83–162	228–1140	92	127	94–172	254–1270	70	186	135–276	372–1860
10	87	166	125–230	332–1660	81	108	78–149	216–1080	45	32	20–54	64–320	74	164	116–239	328–1640
11	57	8	4–23	16–80	87	247	183–346	494–2470	46	53	34–83	106–530	147	47	32–72	94–470
12	74	115	83–159	230–1150	86	174	129–246	348–1740	45	132	84–253	264–1320	118	251	195–331	502–2510
13	73	125	90–174	250–1250	105	243	186–327	486–2430	90	195	144–270	390–1950	87	187	138–256	374–1870
14	55	90	61–132	180–900	70	179	129–278	358–1790	67	123	88–178	246–1230	81	80	56–115	160–800
15	87	123	92–169	246–1230	81	175	128–240	350–1750	77	202	148–293	404–2020	92	140	104–189	280–1400

For each patient, the number of miracidia genotyped (n), the estimated effective number of breeders (N_b_) and 95% confidence interval (CI) are given. For each patient, the schistosome census size (N_c_) is estimated using an N_b_/N_c_ ratio of 0.1 to 0.5.

**Figure 1:**
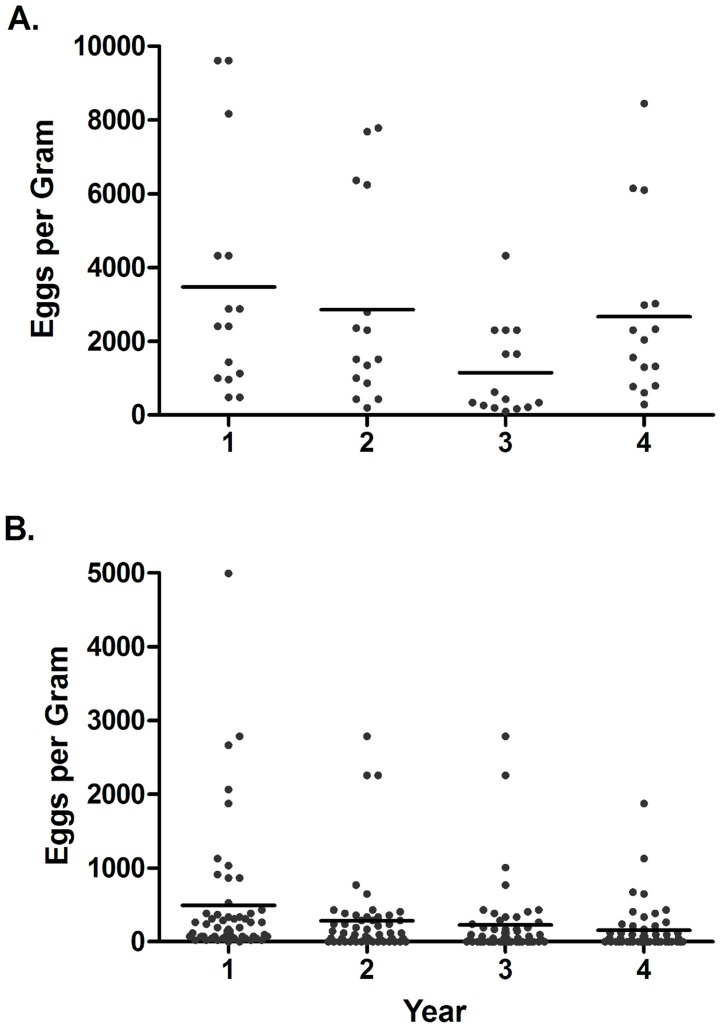
Mean number of schistosome eggs per gram of feces in patients for four years during annual mass praziquantel administration to school aged children. A. 15 patients deemed “phenotypically susceptible” to schistosomiasis during MDA from which genetic samples were collected. Differences were due to a decrease in prevalence rather than a reduction in egg counts in infected individuals B. 52 randomly sampled children. Note the difference in scale of the Y-axis of both figures as egg burdens were much higher in the phenotypically susceptible group.

**Figure 2:**
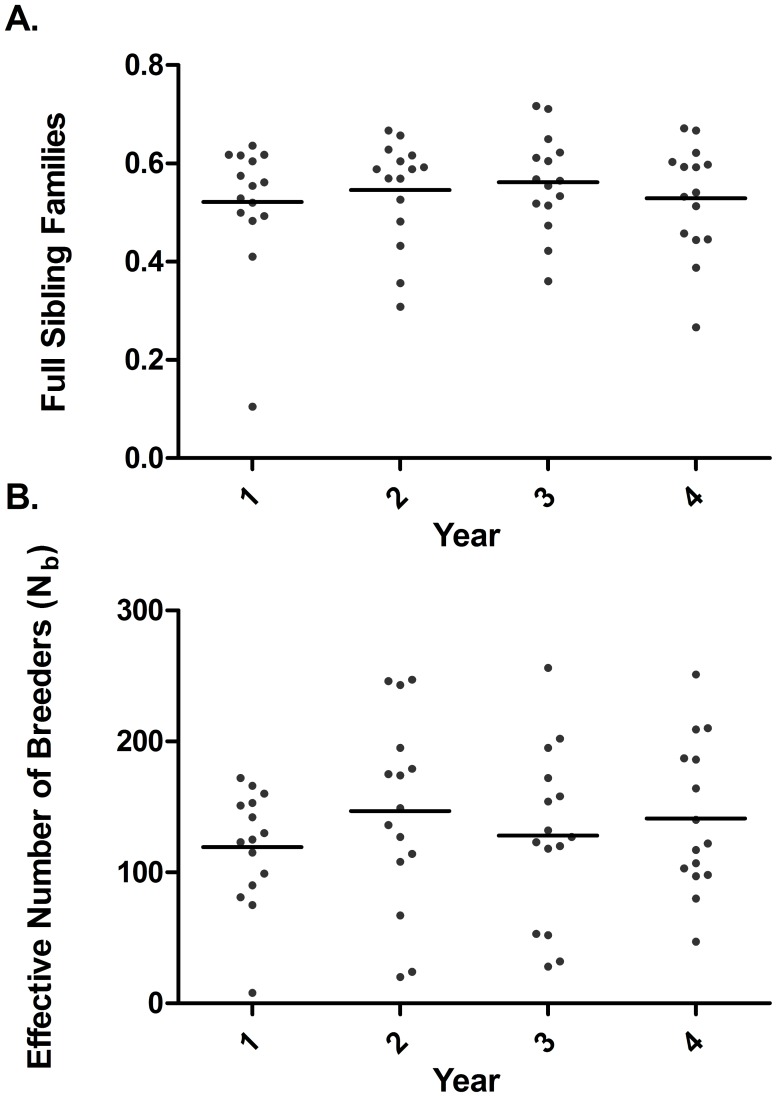
Mean changes in genetic estimators of schistosome worm burdens collected from humans during four years of an annual mass treatment program. Lines indicate means. A. number of full sibling families standardized according to sample size. B. effective number of breeders as estimated using the sibling assignment method.

Repeated measures ANOVA of the 15 phenotypically susceptible children indicated that egg counts were significantly different among years (F_3,42_ = 5·662, P = 0·002) (Fig. 1A). Prevalence of infection in all 67 patients declined significantly from 97% at year one level, to 77.6% in year 2, and 68.7% in years 3 and 4 (Fisher exact test Yr1 v. Yr 4, P = 0·0001) (Fig. 1B). After the first year, three individuals were not found infected in any subsequent years, and three additional children remained negative for the remainder of the study after treatment in the second year. Polynomial contrasts indicated significant second and third degree trends (quadratic and cubic) (F_1,14_ = 8·012, P = 0·012, F_1,14_ = 7·014, P = 0·014), but not a significant linear trend (first degree) (F_1,14_ = 1·183, P = 0·183) (Fig. 2A). The trend was a decline in egg counts through years 1, 2, and 3 and then an incline back to original levels in year 4. Egg counts in the remaining 52 children also changed significantly over time (F_3,153_ = 9·89, P<0.001) (Fig. 2B) with a significant linear trend (F_1,51_ = 39·562, P<0.001), but not quadratic or cubic trends (F_1,51_ = 1·8, P = 0·176; F_1,51_ = 0·005, P = 0·946). The linear trend was a decline over the four years. This change appeared to be driven by an increase in uninfected individuals (egg counts of 0) rather than a decrease in egg counts of infected individuals, as indicated by the decline in prevalence) because a one-way ANOVA using only values from infected individuals indicated no significant differences between years (F_3,145_ = 1·019, P = 0·604). Thus, fewer children were infected; however, those that became reinfected did not have significantly lower burdens.

**Figure 3:**
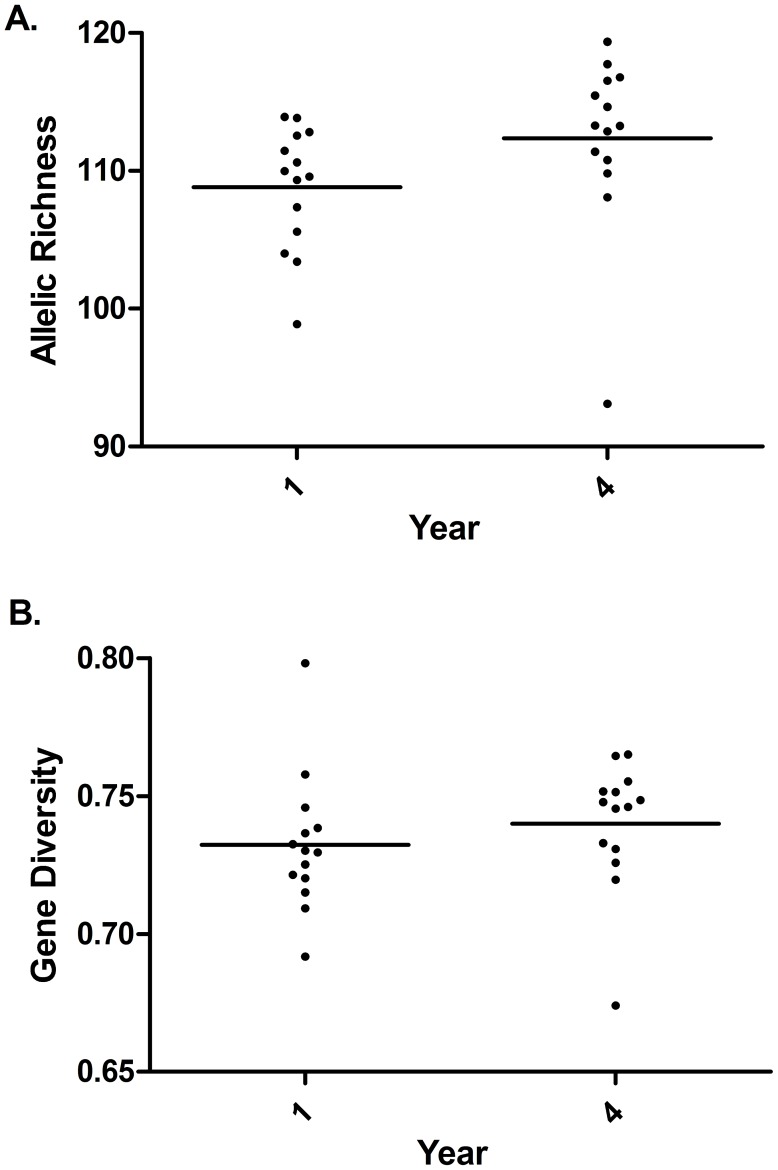
Mean changes in genetic diversity of schistosomes collected from humans before and after four years of an annual mass treatment program. Lines indicate means. Data are corrected for sibling structure. A. allelic richness B. gene diversity.

The number of full sibling families and effective number of breeders did not show statistically significant change over time (full sibling families: F_3,42_ = 0·397, P = 0·756; N_b_: 0·757, P = 0·757) (Figure 3). Allelic richness and gene diversity significantly increased over time (allelic richness: F_1,126_ = 416·2, P<0.0001; gene diversity: F_1,126_ = 123·5; P<0.0001). Significant interactions were detected between patient and time for both parameters (allelic richness: F_13,126_ = 159·5; P<0.0001; gene diversity: F_13,126_ = 159·5; P<0.0001) indicating that for some patients, these values decreased over time.

## Discussion

The findings of our study highlight the ability of schistosomes to remain stubbornly entrenched in endemic areas despite school based MDA. Over a period of four years, we found a reduction in the number of children infected with schistosomiasis, a primary goal of the JICA/KEMRI program. This program has undoubtedly reduced or prevented severe morbidity caused by schistosome infection in children. However, we have found no evidence of an overall reduction of schistosome transmission in the region either by monitoring worm burdens via either egg counts or using genetic parameters to monitor population changes. We saw changes in egg counts in our 15 phenotypically susceptible patients over time, but this change was a decline in years 1–3, but one that was followed by an increase back to initial levels in year 4. Also, in the other 52 children, although some did not become reinfected at some of the sampling times which is certainly a desired outcome of control, we saw no change in the egg burdens over the four years of study for these individuals when they did reacquire infections. Furthermore, among our 15 phenotypically susceptible children, we detected an increase in genetic diversity over the course of treatment, which would not be expected had a population bottleneck or significant decline actually occurred due to treatment. Increased diversity is unlikely to be explained by selection due to the MDA program because these microsatellite markers are presumably neutral with regard to drug resistance or immune evasion. The mechanism driving the increase in genetic diversity is unknown and could be due to population increase (more individuals means more chance of novel mutations), or immigration from other surrounding schistosome populations (outbreeding). Although annual treatments of all school aged children in the Mwea region has been successful in reducing infection in children, it appears to have had no effect on the overall transmission of schistosomes in the region. This observation is consistent with that of Kihara et al. [26] who observed that following each round of treatment, prevalence increased, albeit not to baseline levels. Note that our sampling did not include a baseline measure.

Interestingly, annual prevalence in the 67 children was seen to decline over time, but this decline was not matched in the egg counts of infected individuals. Following MDA, prevalence is predicted to rapidly rise to pre-control levels, while mean worm burden rises much more slowly due to the highly non-linear relationship between prevalence and intensity (large changes in intensity result in very small changes in prevalence) [34]. This pattern may be reflecting acquired resistance after repeated drug treatment or behavioral changes as the children age and due to education. Another explanation is a lack of reliability of the Kato Katz technique to accurately measure worm burdens [18], [35], [36]. Understanding the reasons behind these changes is important for successful monitoring and also for successful modeling of MDA strategies and should be further investigated.

Our findings differ from recent studies that detected a reduction in genetic diversity (allelic richness and gene diversity) of schistosomes collected from fecal samples of children enrolled in a school based treatment program near Lake Victoria in Tanzania [10], [21]. In these studies, a reduction in allelic richness and gene diversity (heterozygosity) was found between baseline infection levels and a single year following treatment. The differences between these studies and ours may indicate important differences in local epidemiology, MDA programs, or monitoring protocols. For instance, sampling schemes differed greatly between our studies. We sampled over four years rather than between baseline and year 1 which gives a longer perspective on population changes.

Our modest sample size of patients did not appear to limit statistical power to detect deviations from the null hypothesis of no change among years as we were able to reject the null hypothesis in many of our analyses. The patterns we found did not fit the hypothesized decline over time. Also, in a previous study of schistosome genetic diversity, resampling simulations indicated that data from only 10 hosts are enough to achieve sufficient statistical power to detect differences in genetic diversity [10]. Here we note that caution may be required in extrapolating our results for these15 children to the schistosome population in the entire Mwea scheme area. However, these children come from different schools within the scheme which varied in their baseline prevalence and level of transmission. From our results, we are confident that large declines in the schistosome population, such as those nearing elimination, were not occurring in the Mwea population. Due to costs, a tradeoff between in depth sampling, number of individuals, and number of years to sample is inevitable. By choosing an increased depth of sampling over four years, we were able to look at longer term patterns of change, use novel measures of population change (N_b_ and FSF), and more accurately measure genetic diversity after correcting for bias that is present when collecting miracidia from a patient.

Our findings coincide with recent modeling results that indicate deworming programs targeted solely at school aged children are likely to be limited in terms of their impact on community-wide parasite transmission even at high levels of drug efficacy (95%) and coverage of school aged children (85%) because of the relatively small portion of the parasite population exposed to treatment [37]. Increasing the demographic that is included in the treatment program is likely to reduce parasite transmission; however, it must also be noted that increasing the proportion of parasites that are exposed to drug treatment is also predicted to increase the selection pressure on drug resistance [38]. This is a particular concern for schistosomiasis given that there is only one drug effective against all schistosomes and that schistosomes with reduced susceptibility to praziquantel have been reported from Kenya [39]. Together, these findings underscore the need to include alternative control approaches [34], [40]. In general, in our view, control efforts should take cognizance of the specific local environmental and epidemiological circumstances, try to use the inherent biodiversity present to limit transmission, and use an integrated multi-pronged approach as a more sustainable way forward.

In summary, after four years of school based MDA, we did not detect a reduction in schistosome population. Our data set is unique in that it combines both egg counts and novel genetic parameters to measure population changes. Also, our study follows a portion of the population that has remained phenotypically susceptible to monitor changes in the schistosome population over a four year period with annual school-based mass treatments. This design allows changes to be monitored without interference of acquired immunity and accumulation of worms over time, two road blocks to measuring the efficacy of school-based treatment programs on a community wide scale.

## Supporting Information

Checklist S1 STROBE Checklist.
(DOC)
